# Caregivers’ Role in the Effectiveness of Two Dutch School-Based Nutrition Education Programmes for Children Aged 7–12 Years Old

**DOI:** 10.3390/nu13010140

**Published:** 2021-01-01

**Authors:** Angeliek Verdonschot, Emely de Vet, Natalie van Seeters, Jolieke Warmer, Clare E. Collins, Tamara Bucher, Annemien Haveman-Nies

**Affiliations:** 1 Consumption and Healthy Lifestyles Group, Wageningen University & Research, 6700 EW Wageningen, The Netherlands; emely.devet@wur.nl (E.d.V.); nvs_pien@hotmail.com (N.v.S.); joliekewarmer@upcmail.nl (J.W.); annemien.haveman@wur.nl (A.H.-N.); 2 Priority Research Center for Physical Activity and Nutrition (PRCPAN), The University of Newcastle, Callaghan, NSW 2308, Australia; clare.collins@newcastle.edu.au (C.E.C.); tamara.bucher@newcastle.edu.au (T.B.); 3 School of Health Sciences, Faculty of Health and Medicine, The University of Newcastle, Callaghan, NSW 2308, Australia; 4 School of Environmental and Life Sciences (SELS), Faculty of Science, The University of Newcastle, Callaghan, NSW 2308, Australia

**Keywords:** home environment, nutrition education programmes, caregivers, FV intake, primary school children

## Abstract

Childhood eating behaviours can track into adulthood. Therefore, programmes that support early healthy eating, including school-based nutrition education programmes, are important. Although school-based programmes may be beneficial in improving nutrition knowledge, impact on actual fruit and vegetable (FV) intake is generally limited as FV intake is also influenced by the home environment. The current study includes secondary analyses of data from an evaluation study on Dutch nutrition education and examined the role of caregivers’ health promotion behaviours (HPB) in influencing healthy eating behaviours in primary school children (*n* = 1460, aged 7–12 years) and whether caregivers’ HPB contribute to programme effectiveness. Children’s nutrition knowledge, FV intake and caregivers’ HPB (FV/sugar-sweetened beverages/sweets provision to take to school, cooking together and talking about healthy food at home) were measured by child-reported questionnaires at baseline, during, and 6 months post-programme. Results indicated that caregivers’ HPB was positively associated with children’s healthy eating behaviours and that programme effectiveness was highest in those in the lower HPB subcategory. In conclusion, children with less encouragement to eat healthily at home potentially benefit more from school-based nutrition education programmes than children receiving more encouragement. This highlights the important role of the home environment in supporting healthy eating behaviour in children.

## 1. Introduction

It is important to optimise eating patterns in early life since eating behaviours that develop during childhood are likely to track into adulthood [1]. Higher fruit and vegetable (FV) intake as a component of healthy eating habits helps to lower the risk for obesity, cardiovascular disease and certain types of cancer [2]. Therefore, nutrition education programmes targeting children are expedient, with the promotion of FV intake through nutrition and health policies recommended [3].

Schools are an ideal setting for the promotion of healthy eating since children from various socioeconomic backgrounds can be reached [4]. Hence, worldwide many school-based nutrition education programmes are developed and evaluated. A systematic review by Evans et al. (2012) found that such school-based interventions only moderately improve children’s fruit intake (mean improvement of 0.24 portions, 95% CI: 0.05, 0.43 portions) and often fail to increase children’s vegetable intake (mean improvement of 0.07 portions, 95% CI: −0.03, 0.16 portions) [5].

Caregivers have a prime impact on the development of their children’s attitudes towards food, choices made when selecting foods, preparation and timing of meals, with encouragement to eat FV in establishing healthful dietary patterns [6,7,8]. Previous research described a health-promoting home environment as an environment where FVs are available, caregivers are positive role models and where children are encouraged to eat FV and is positively associated with FV intake in children [9]. For example, when FV are available, children are more likely to eat FV [10]. In addition, children also learn about eating by observing other people’s behaviours [11]. Previous research found that children’s FV intake was positively related to caregivers’ FV intake, indicating the role modelling function of caregivers [12]. The frequency of eating together as a whole family is positively associated with consumption of healthy foods such as FV, grains and calcium-rich foods, and negatively associated with consumption of sugar-sweetened beverages (SSBs) [13,14]. A recent review found a positive association between child involvement in the preparation of home meals and their FV intake [15].

In addition, feeding styles used by caregivers within the home environment that are used to maintain or modify children’s eating behaviours contribute to children’s dietary intake. Baumrind [16] and Maccoby and Martin [17] described four child-feeding styles: (1) authoritarian (e.g., restricting the child from eating desserts), (2) permissive (e.g., the child is allowed to eat whatever he or she wants in whatever quantities he or she wants), (3) authoritative (a balance between authoritarian and permissive, e.g., the child is encouraged to eat healthy foods but has some choice to eat other foods as well) and (4) neglective (characterised by uninvolved caregivers, e.g., the child is completely free to maintain eating habits without any concern of the caregivers) [16,18,19,20]. Authoritarian feeding practices are associated with pressuring a child to eat, restrictive parental food behaviours [21], lower availability of FV [22] and lower intakes of FV and juices [23], whereas permissive feeding is inversely related to monitoring of child dietary intake [21] and associated with drinking less milk and lower consumption of all nutrients except fat [24,25]. Authoritative feeding is associated with parental monitoring of child food intake [21] and higher FV intake, FV availability and lower consumption of unhealthy foods [22,26]. Another promising strategy that was identified to encourage children to consume FV is providing children with choice within healthy food options, such as offering two types of vegetables during dinner [27,28]. Lastly, neglectful feeding practices are associated with lower fruit consumption and lower attitude, subjective norm, social support, modelling, self-efficacy, and intention towards eating fruit [29]. 

Although the importance of caregivers in the development of healthy eating behaviour in children is acknowledged in most nutrition education programmes, only limited, or nonactive involvement of caregivers within the school environment is included. In addition, the active engagement of the home environment is often not taken into consideration [30,31]. Examples of nonactive caregiver involvement within such school-based programmes include receiving information through newsletters, folders or homework assignments. In contrast, active involvement contains more experiential learning behaviours such as cooking together (children and caregivers at school or at home), talking about healthy eating lessons learned at school or FV provision by caregivers [31,32,33]. A recent systematic review by Morgan et al. (2020) [30] assessed the effects of caregiver involvement in interventions for improving children’s dietary intake and physical activity behaviours based on 23 randomised controlled trials and concluded that there is not enough evidence to confirm added value of involving caregivers in health-promoting interventions. This lack of evidence was mainly due to the methodological limitations of these studies [30].

In European countries like the Netherlands, most children take their own snacks, drinks and lunch from home to school, or have lunch at home, as generally no school meals are offered [34]. This indicates the importance of involving the home environment in supporting healthy eating in children, as caregivers decide what items to purchase at the supermarket and then give their children to take to school. The effectiveness of Dutch school-based nutrition education programmes on children’s healthy eating behaviour is therefore potentially more dependent on health promotion behaviour of caregivers, compared to other countries where snacks, lunch and drinks are provided by the school. Moreover, school-based nutrition education may be redundant if caregivers already ensure their children’s diet is healthy. However, it currently remains unclear how caregivers’ health promotion behaviours influence the results of school-based nutrition programmes. Having a better understanding of this influence may contribute to the enhanced design and effectiveness of future programmes.

Therefore, the current study aimed to address the following research questions: (1) what is the association between active health promotion behaviour of caregivers within the home environment and children’s FV consumption and nutrition knowledge? and (2) what is the contribution of active health promotion behaviour of caregivers to the effects of nutrition education programmes on children’s FV intake?

## 2. Materials and Methods

The current study is a secondary analysis of data from an evaluation study. The study details and results of both programmes on children’s nutrition knowledge and FV consumption and school characteristics (size, principle and school food policies) are described elsewhere [35]. The study included 37 primary schools and 1460 children aged 7–12 years old, allocated to three study groups: (1) the ‘FV + Ed group’, schools (*n* = 15) that implemented the FV provision programme (EU-Schoolfruit [33]) and the Education programme (Taste Lessons [32]), (2) the ‘FV group’ including schools (*n* = 12) that implemented only the FV provision programme and (3) schools (*n* = 10) that did not implement either programme (control group). A description of the programmes can be found in Appendix A.

### 2.1. Measures

#### 2.1.1. Primary Outcome Measures

A self-reported (hardcopy) questionnaire was used to collect primary outcome measures pre-intervention (baseline, T0), during the intervention (approximately 6 months after baseline, T1) and 6 months post-intervention (approximately 12 months after baseline, T2). Items were based on previous comparable studies about nutrition education [36,37,38,39]. The following three primary outcome measures were collected: (1) children’s nutrition knowledge, (2) children’s FV intake and (3) caregivers’ health promotion behaviour. 

##### Nutrition Knowledge

Children’s nutrition knowledge was measured via 24 questionnaire items related to the content of the education programme (Taste Lessons) adapted from Vereecken et al. (2012) [37]. Different from the original questionnaire, an ‘I don’t know’ option was added to the response options. In addition, the questionnaire was complemented with items on senses, recommended portion sizes and food production, themes related to the content of Taste Lessons and based on a previous effectiveness study of Taste Lessons [36]. Correct answers scored 1 point, while incorrect and ‘I don’t know’ responses received 0 points. The nutrition knowledge score was the sum of all items divided by the number of items answered.

##### Fruit and Vegetable Intake

Children’s FV consumption was measured through a validated 24-h recall, described elsewhere [38]. As children had to report their FV intake from the previous school day, the questionnaire was completed on a weekday with the exception of Monday. Similar to Haraldsdóttir et al. (2005), the 24-h recall consisted of three-time intervals: morning, afternoon and evening. The children had to fill in a precoded table, specifying the type and amount of FV. The table included open spaces for FV consumed that were not listed. Juices, smoothies, nuts, legumes and potatoes (except sweet potato) were excluded, as they are not part of the fruit and vegetable group, based on the Dutch healthy guidelines (in Dutch: ‘De Schijf van Vijf’) [40]. The reported portion sizes were converted into grams based on Dutch standard portion sizes [41]. If the amount or type of FV was not reported or unclear, the average amount and most common type was used, according to the Dutch National Food Consumption Survey [42]. To calculate vegetable percentages in mixed dishes or soups, the online Dutch nutrients database was used (in Dutch: ‘Nederlands Voedingsstoffenbestand’ (NEVO)) [43]. 

##### Health Promotion Behaviour

Caregivers’ health promotion behaviour (HPB) was measured through five items. The first four items asked about the frequency of the provision of (1) FV, (2) sweets and (3) sugar-sweetened beverages (SSBs), and (4) children helping with cooking at home (Table 1). The items on provision behaviour of the caregivers were related to the morning snacks and drinks the children received from their caregivers to take to school. Answering categories were ‘every day’, ‘3–4 times a week’, ‘2–3 times a week’, ‘once a week’, ‘sometimes’ (only for cooking item) and ‘never’. The fifth item asked children if they talked about healthy eating in their home environment, with answer categories: ‘no’, ‘sometimes’ or ‘yes’. The two items on the provision of sweets and SSBs were reverse coded first, to be in line with the other items, indicating a high score is related to high HPB, and a low score is related to low HPB (i.e., consuming SSBs and sweets is related to an unhealthy diet and consuming FV is related to a healthy diet). Subsequently, the HPB results were categorised in a ‘low HPB’ and ‘high HPB’ group by combining answer categories. ‘Low HPB’ indicates children with caregivers who scored low in HPB (e.g., providing sweets or SSBs ranging from every day up to 2–3 times a week), and ‘high HPB’ indicates children with caregivers who scored high in HPB (e.g., providing sweets or SSBs ranging from never up to 1–2 times a week, and for FV provision the other way around). The item on talking about healthy eating was divided into three categories corresponding with the three answer categories (‘no = low HPB’, ‘sometimes = medium HPB’ and ‘yes = high HPB’).

#### 2.1.2. Other Measures

During the first measurement (baseline, T0), data on participating children’s age (in years), sex and grade (6 or 7) were reported through the questionnaire.

### 2.2. Statistical Analysis

Multilevel linear models were used to measure the effectiveness of the programmes on children’s nutrition knowledge and FV intake. Details about this evaluation study are described in more detail elsewhere [35]. To answer RQ1 ‘What is the association between active health promotion behaviour (HPB) of caregivers and children’s FV consumption and nutrition knowledge?’, the baseline results of children’s FV intake and nutrition knowledge were evaluated based on means and standard deviations (SD), for the five variables on caregivers’ HPB. Subsequently, multilevel regression analyses were conducted including three levels: (1) student, (2) class and (3) school. HPB was added to the model as a moderator to measure the contribution of caregivers’ HPB to the effects of the two nutrition education programmes (FV provision and Education) on children’s FV intake (RQ2). Change in children’s FV intake in short- and long-term among the five HPB variables were evaluated by comparing baseline results (T0) with the second (T1) and third measurement (T2). A *p*-value of less than 0.05 was considered to be significant. The analyses were performed using statistical software R, version 3.6.1 [44] including packages ’car’ and ’nlme’.

## 3. Results

### 3.1. Caregivers’ HPB and Children’s FV Intake and Nutrition Knowledge

Table 2 reports mean child FV intake and nutrition knowledge at baseline, for the categories of the caregivers’ HPB. More than half of the children reported they received FV every day or 3–4 times a week from their caregivers to take to school (65%) and relatively few children indicated they received FV from home 2–3 times or once a week, or never (35%). For the provision of sugar-sweetened beverages (SSBs), more than half of the children reported never or once a week receiving SSBs from their caregivers to take to school (52%), but also many children indicated they receive SSBs on a daily base (30%). More than half of the children reported never or once a week receiving sweets from home (55%), and relatively few children listed they received sweets 3–4 times per week, or everyday (24%). More than half of the children reported they help their caregivers with cooking at home ‘sometimes’ (54%), with ‘3–4 times a week’ answered least often (6%). In line with the results of ‘helping with cooking’, more than half of the children reported they sometimes talk about healthy food with their caregivers at home (54%), and the ‘yes’ and ‘no’ answers for this item were relatively equal indicated by the children (yes: 24%, no: 22%).

The association between caregivers’ HPB and FV intake and nutrition knowledge in children indicates that children who receive FV frequently from home to take to school reported a significantly higher FV intake than children who receive FV less frequently (Table 2). This positive association was also found for children’s nutrition knowledge. In line with these findings, children who received less frequently sweets or SSBs from home (never or 1/week) reported a higher FV intake and nutrition knowledge, compared to children who received sweets every day or SSBs from home to take to school. 

Further, children who helped with cooking at home more often reported higher FV consumption and higher nutrition knowledge, compared to children who infrequently helped with cooking. Similar results were found regarding children’s conversations about healthy eating with their caregivers, indicating a positive association between talking about healthy eating and children’s FV intake and nutrition knowledge.

### 3.2. Contribution of Caregivers’ HPB to the Effectiveness of the Programmes

**Figure 1:**
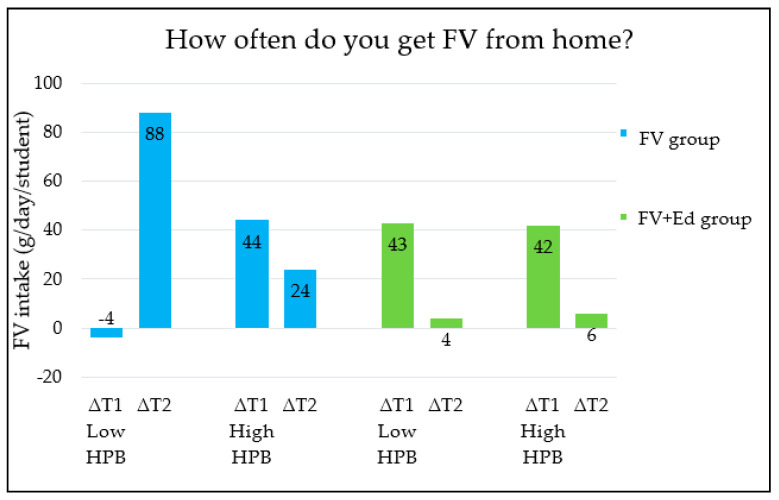
Differences in children’s FV intake, stratified by caregivers’ HPB in FV- and FV + Ed group, compared to control group—FV provision. HPB = caregivers’ health promotion behaviour, ∆T1 = Difference between T0 and T1, ∆T2 = Difference between T0 and T2.

**Figure 2:**
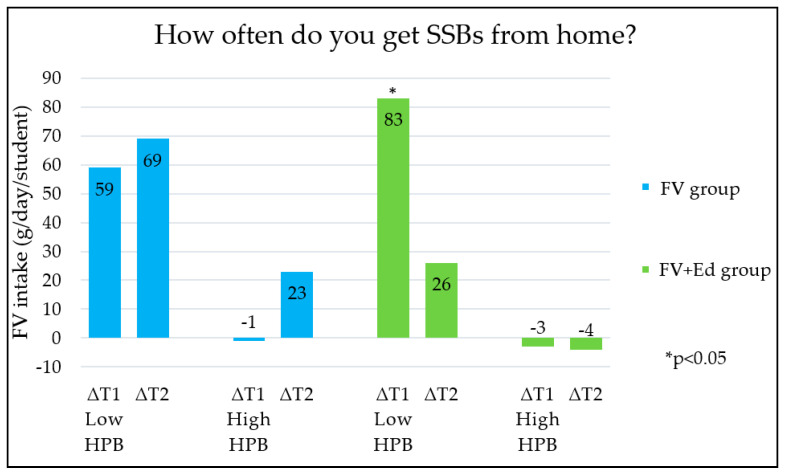
Differences in children’s FV intake, stratified by caregivers’ HPB in FV- and FV + Ed group, compared to the control group—SSBs provision. HPB = caregivers’ health promotion behaviour, ∆T1 = Difference between T0 and T1, ∆T2 = Difference between T0 and T2.

**Figure 3:**
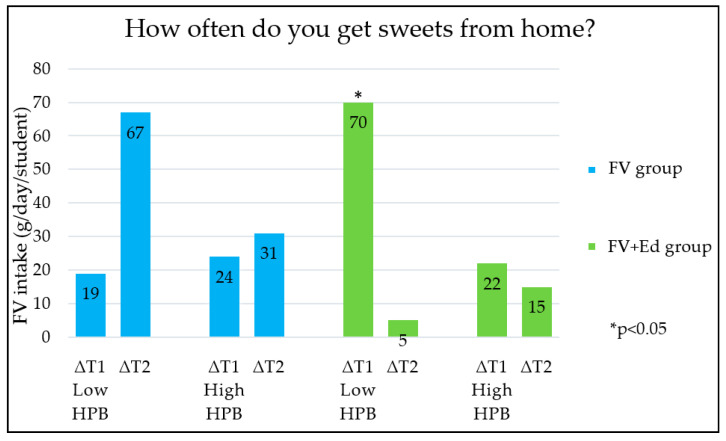
Differences in children’s FV intake, stratified by caregivers’ HPB in FV- and FV + Ed group, compared to the control group—Sweets provision. HPB = caregivers’ health promotion behaviour, ∆T1 = Difference between T0 and T1, ∆T2 = Difference between T0 and T2.

**Figure 4:**
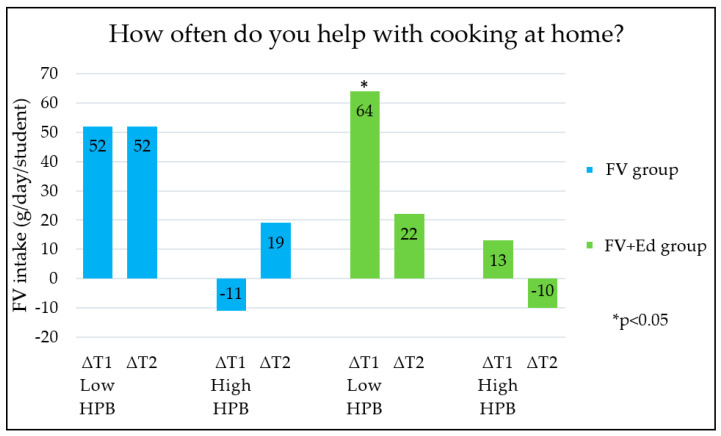
Differences in children’s FV intake, stratified by caregivers’ HPB in FV- and FV + Ed group, compared to the control group—Cooking together. HPB = caregivers’ health promotion behaviour, ∆T1 = Difference between T0 and T1, ∆T2 = Difference between T0 and T2.

**Figure 5:**
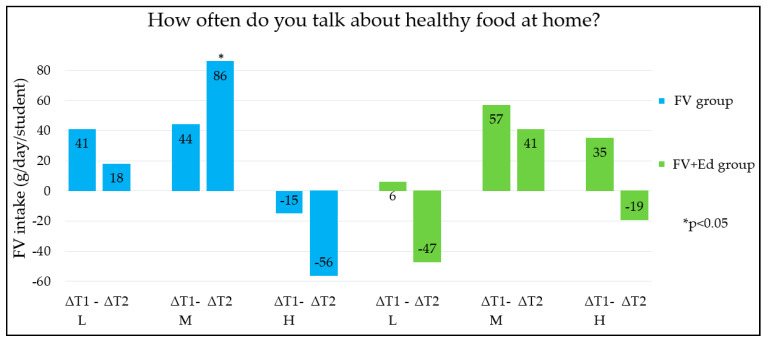
Differences in children’s FV intake, stratified by caregivers’ HPB (L = low HPB, M = medium HPB, H = high HPB) in FV- and FV+Ed group, compared to the control group—Talking about healthy food. HPB = caregivers’ health promotion behaviour, ∆T1 = Difference between T0 and T1, ∆T2 = Difference between T0 and T2.

Changes in children’s FV intake between the three measurements (T0, T1 and T2) were different for the groups categorised by HPB (low/medium/high HPB). The five HPB categories, each measured by an individual question in the questionnaire (Table 1) are shown in Figure 1, Figure 2, Figure 3, Figure 4 and Figure 5 (∆T1 = difference between T0 and T1, ∆T2 = difference between T0 and T2). Considering the first HPB item on FV provision (based on the question ‘How often do you get FV from home to take to school?’), no differences in programme effectiveness were found in FV intake for children with caregivers who report low and high HPB at T1 and T2, compared to the control group (Figure 1). For the provision of sugar-sweetened beverages, sweets and cooking together, a significant difference in FV intake in short term was observed in the FV + Ed group between children of caregivers with low HPB compared to children of caregivers with high HPB, but not in the long term (Figure 2, Figure 3 and Figure 4). Regarding ‘talking about healthy food’, no effect of HPB on programme effectiveness was identified, with the exception of a significant increase in FV intake in the middle HPB category for the FV group in the long term, compared to the control group (Figure 5).

## 4. Discussion

The aim of the current study was to investigate caregivers’ health promotion behaviour (HPB) in relation to children’s FV intake and nutrition knowledge and effectiveness of two Dutch nutrition education programmes. Firstly, caregivers’ HPB was positively associated with children’s FV intake and nutrition knowledge at baseline, suggesting that support from caregivers in healthy eating behaviour (e.g., providing FV to take to school) improves children’s healthy eating behaviour (e.g., consuming more FV). This is in line with previous literature indicating positive associations between home availability, family rules and caregivers’ encouragement and children’s FV intake [9]. 

When compared to children in the high HPB group, FV intake increased significantly in the short term for children who participated in both programmes and received regular sweets, SSBs or who helped less often with cooking at home (low HPB group). This suggests that nutrition education programmes are especially effective in increasing FV consumption for children who need the most support (low HPB). Considering FV provision and talking about healthy eating, no trend for impact on FV intake was observed.

### 4.1. The Association between Caregivers’ HPB and Children’s Healthy Eating Behaviour

Current results indicate that less than half of the children received FV every day from their caregivers to take to school and that sweets are not provided often, which is supportive of healthy eating in children. This may be related to the fact that most Dutch schools adopt policies that regulate unhealthy food and/or drinks brought to school from home and support consumption of FV in the morning breaks [34,35]. However, the results of the current study show there is room for improvement, given regular consumption of SSBs and unhealthy snacks at school are still reported. For example, almost one-third of the children reported that they receive SSBs to take to school on a daily basis. This may be due to caregivers’ unawareness of the importance of a healthy diet, or lack of attention to HPB in school as described in previous literature [45]. 

To answer our first research question, a positive association between caregivers’ health promotion behaviour and children’s FV intake and nutrition knowledge was found. Our results indicate that children who receive FV more often to take to school, eat more FV during the day. This supports previous research showing a positive association between the home food environment and children’s diets [9,46,47,48].

Regarding caregivers’ HPB and children’s nutrition knowledge, limited literature is available as children’s healthy eating behaviour (such as FV intake) is mostly addressed as the main outcome, instead of nutrition knowledge. Previous research found a positive association between caregivers’ nutrition knowledge and children’s nutrition knowledge [49] and dietary intakes [50]. Similar results were found in a different study, indicating a positive correlation between mothers’ and children’s nutritional knowledge and fruit consumption [51]. This relationship may be explained by caregivers’ HPB, but no firm conclusions can be drawn. 

Furthermore, considering caregivers’ SSBs provision and sweets provision, caregivers’ less frequent provision was associated with higher FV intake and nutrition knowledge in children. No literature was located on SSBs or sweets provision by caregivers to take to school in relation to FV intake and nutrition knowledge. However, the literature on SSBs intake and fruit consumption found similar results, indicating children who drink SSBs most often, eat daily 0.5 portions of fruits less, compared to children who rarely drink SSBs [52]. This association was also reported in a study of Marshall et al. (2013), which found that consuming SSBs was associated with lower intake of multiple nutrients (e.g., vitamin B-6 (−0.20 of Adequate Ratio (AR)), magnesium (−0.25 AR) and iron (−0.25 AR)) and overall diet quality [53]. Also, a meta-analysis, conducted by Vartanian et al. (2006) found clear associations between higher SSBs intakes and lower nutrients intakes [54]. This can be explained by the fact that most SSBs are energy-dense and nutrient-poor, indicating that consuming more SSBs may displace nutrient-dense foods such as fruits and vegetables. In addition, based on a systematic review and meta-analysis (95 studies), FV consumption is associated with reduced risk of cardiovascular diseases, cancer and all-cause mortality and contributes to health, which is the main goal of nutrition education programmes [55]. Despite these findings, there is still a need for further research on the provision of SSBs and sweets by caregivers and children’s FV intake and nutrition knowledge to further confirm this association.

The current study results indicate that children who helped with cooking at home more often reported a higher FV intake and level of nutrition knowledge than children who do not often help with cooking. These findings are supported by the literature, where several studies show that children helping with cooking in the home environment is associated with a higher FV consumption [56,57,58]. One of these studies reported that children who help with cooking on a daily basis, eat approximately a portion fruit or vegetable more each day, compared to children who never help with cooking [57]. In the current study, a difference of 136 g/day/student was found between these groups, which corresponds to about 1.7 servings of fruits or vegetables, based on Dutch portion sizes [41]. No literature was found on the association between cooking at home and nutrition knowledge.

Regarding the question of ‘talking about healthy eating in the home environment’, baseline results of the current study found a positive association with FV intake and nutrition knowledge in children. This is in line with previous research indicating that talking about healthy eating at home is associated with higher FV consumption in children [59,60] and a study that found increased nutrition knowledge about the ‘5-a-day of fruits and veggies intake’ as a result of nutrition education using caregivers involvement [61]. This may be explained by the fact that it is likely that caregivers found healthy eating more important if they talk about it with their children, resulting in healthier behaviour (e.g., by providing more FV (FV intake), or explaining nutrition/health-related issues (nutrition knowledge)), compared to caregivers who do not talk about it with their children. 

### 4.2. The Contribution of Caregivers’ HPB to the Effectiveness of the Programmes

Caregivers’ health promotion behaviours contribute to the effectiveness of the two nutrition education programmes on children’s FV intake, especially in children who are less supported to eat healthily at home (low HPB) (RQ2). FV provision by caregivers (to take to school) did not significantly influence the effectiveness of the programmes. This nonsignificant result may be explained by the fact that most of the intervention schools have participated in the FV provision programme in the previous two years (26 out of the 27 intervention schools participated in the FV programme in the school year 2017–2018 and 14 participated in 2016–2017), which may influence caregivers’ FV provision as they may provide less FV since their child already receives FV at school (via the FV provision programme). In contrary, the FV provision programme may also encourage caregivers to provide FV, as they may become more aware of the importance of consuming FV (e.g., via talking about it with their child) or follow the suggestions made by the school. Also, caregivers’ behaviour may be influenced by school-based nutrition education, for example, when their child wants to eat FV and ask their caregivers to buy it. Therefore, future research on caregivers’ FV provision behaviour, while controlling for a potential influence of FV provision programmes in previous years are recommended. 

FV intake changes were greatest in the short term (∆T1) in children who received SSBs or sweets to take to school more frequently or cooked at home together less often (low HPB). This means that nutrition education programmes seem to have stronger beneficial effects in children who are less supported generally to eat healthily within their home environment. This may be explained by the fact that healthy eating behaviour in children who are less supported to eat healthily by their caregivers (low HPB) have more room for improvement, compared to children who are already supported to eat healthily (high HPB) and likely already have a healthy diet. In addition, the fact that the FV provision programme was active during the second measurement (T1) and was not running anymore during the last measurement (T2) may have influenced the results. 

The results of the current study indicate there is an association between caregivers’ HPB and the effectiveness of nutrition education programmes, but further clarification is required given firm conclusions cannot yet be drawn due to the complexity of this concept, the influence of the food provision programme in previous years on caregivers behaviour, the methodological limitations and lack of data [30]. Our results may be seen as a starting point for evidence of the important role of caregivers in supporting healthy eating in children and provides some insights that may contribute to the development of future effective programmes.

### 4.3. Strengths and Limitations

Strengths of the current study were the large sample size of 1392 children and the use of a quasi-experimental design, including a control group, making it likely that changes in FV intake and nutrition knowledge in children could be attributed to the programmes. In addition, the questionnaire used was based on previously validated questionnaires on children’s FV intake [38] and nutrition knowledge [36,37]. The current study was conducted in the context of two existing national FV programmes, already implemented by many schools and that will continue being implemented in the future, meaning results will contribute to future programme refinement and development of new health promotion interventions.

The current study also had some limitations. Firstly, schools were not randomly assigned to a study group (FV+Ed, FV or control group) since it was based on either their intention to participate in the programmes or nonparticipation in any nutrition education programme in the previous two school years (control schools). This approach may have caused selection bias and could have impacted the results. Secondly, caregivers’ HPB was assessed using a child self-reported questionnaire. This may have affected the results as the actual behaviour of caregivers may deviate from the caregiver behaviour as reported by the child. Caregivers’ HPB could only be measured with a limited number of categorical variables measured with single items to enable the children to respond. Therefore, some caution in drawing conclusions is suggested and future research is needed to elaborate on, and to confirm the current study’s findings. Measuring caregivers’ HPB by a questionnaire for the caregivers may lead to more precise HPB estimates. However, conducting research with caregivers has many challenges, such as nonresponses risks, a higher response of the more interested caregivers, which lowers the representativeness, and socially desirable answers.

## 5. Conclusions

Caregivers’ positive health promotion behaviour (HPB) (i.e., encouraging their child to eat healthily) is associated with higher FV consumption and nutrition knowledge in children. Moreover, current results indicate nutrition education programmes are more effective on FV intake in children who have less encouragement to eat healthily in the home environment, compared to children who receive more encouragement. Results highlight the important role of caregivers in supporting healthy eating in children. Future research should be conducted with a more accurate assessment of caregivers’ HPB and programmes targeting a healthier home environment are recommended.

## Figures and Tables

**Table 1:** Variables, number of questions and example of questions and answer options.

Variables	Number of Items	Example Question	Answer Options
Children’s nutrition knowledge	24	‘What is most healthy to drink?’ (images of the products)	(1) Flavored milk, (2) Chocolate, (3) Milk, (4) I don’t know
Children’s FV intake	6	‘What type of vegetable/fruit, and how much did you eat yesterday morning?’	Precoded table with the most common eaten FV and open space to write FV that are not listed
Caregivers’ health promotion behaviour	5	‘How often do you get FV from home to take to school?’	(1) Every day, (2) 3–4 times a week, (3) 2–3 times a week, (4) Once a week, (5) Never
		‘How often do you get sweets from home to take to school?’	(1) Every day, (2) 3–4 times a week, (3) 2–3 times a week, (4) Once a week, (5) Never
		‘How often do you get SSBs from home to take to school?’	(1) Every day, (2) 3–4 times a week, (3) 2–3 times a week, (4) Once a week, (5) Never
		‘How often do you help with cooking at home?’	(1) Every day, (2) 3–4 times a week, (3) 2–3 times a week, (4) Once a week, (5) Sometimes, (6) Never
		‘Do you talk about healthy eating at home?’	(1) Yes, (2) Sometimes, (3) No

**Table 2:** Association between caregivers’ health promotion behaviour (HPB) and children’s FV intake and nutrition knowledge, at baseline (T0).

		Total FV Intake, g/Day/Student		Nutrition Knowledge, Score	
Caregivers’ HPB	*N*^a^ (%)	Mean (SD)	B ^b^	Mean (SD)	B ^b^
FV provision	1382				
Never	164 (12)	214 (220)	ref	2.79 (0.829)	ref
1/week	138 (10)	307 (266)	93 **	2.94 (0.735)	0.15 **
2–3/week	177 (13)	357 (291)	143 **	2.90 (0.818)	0.11 **
3–4/week	269 (19)	333 (272)	119 **	3.10 (0.773)	0.31 **
Every day	634 (46)	357 (257)	143 **	3.04 (0.825)	0.25 **
SSBs provision	1367				
Never	552 (40)	343 (283)	ref	3.03 (0.798)	ref
1/week	156 (12)	402 (249)	59 **	3.01 (0.815)	−0.02
2–3/week	139 (10)	383 (278)	40	3.01 (0.727)	−0.02
3–4/week	110 (8)	350 (274)	7	3.05 (0.827)	0.02
Every day	410 (30)	268 (228)	−75 **	2.91 (0.857)	−0.12 **
Sweets provision	1373				
Never	455 (33)	334 (254)	ref	3.08 (0.816)	ref
1/week	340 (25)	364 (285)	30 *	2.95 (0.781)	−0.13 **
2–3/week	253 (18)	357 (260)	23	2.99 (0.792)	−0.09 *
3–4/week	147 (11)	299 (253)	−35	3.03 (0.789)	−0.05
Every day	178 (13)	255 (262)	−79 **	2.80 (0.876)	−0.28 **
Help with cooking	1374				
Never	157 (11)	261 (231)	ref	2.77 (0.832)	ref
Sometimes	746 (54)	304 (243)	43 *	2.98 (0.833)	0.21 **
1/week	106 (8)	374 (280)	113 **	3.17 (0.663)	0.40 **
2–3/week	130 (10)	414 (303)	153 **	3.31 (0.718)	0.54 **
3–4/week	78 (6)	370 (285)	109 **	3.03 (0.796)	0.26 *
Every day	157 (11)	396 (307)	135 **	2.85 (0.755)	0.08
Talking about food	1377				
No	300 (22)	270 (244)	ref	2.76 (0.818)	ref
Sometimes	747 (54)	326 (264)	56 **	3.00 (0.800)	0.24 **
Yes	330 (24)	396 (275)	126 **	3.16 (0.793)	0.40 **

* *p* < 0.05, ** *p* < 0.01. ^a^ = *N* is number of students; ^b^ = B indicates the difference in FV intake or nutrition knowledge for the HPB variables, compared to the reference (unstandardised).

## Data Availability

The data presented in this study are not publicly available due to privacy and ethical restrictions.

## Appendix A. Description of FV Provision Programme and Education Programme

FV provision programme. EU-Schoolfruit is a Dutch nationwide nutrition education programme for primary schools, developed in 2009 and financed by the European Union. Participating primary schools receive three pieces of fruits and vegetables per week per child for a period of 20 weeks (November-April) to promote FV intake. On average, 3000 Dutch primary schools (45% of all primary schools) participate in this programme every year, reaching approximately 675,000 children annually, based on an average of 225 children per school [33].

Education programme. Taste Lessons, developed in 2006, is another Dutch national school-based nutrition education programme for primary schools that consists of five lessons for each grade, discussing various topics in relation to five themes: ‘taste’, ‘nutrition and health’, ‘cooking’, ‘food production’ and ‘consumer skills’. Each lesson consists of several activities, including experiments, cooking, and tasting. Teachers can implement Taste Lessons during the whole school year. Around 5000 Dutch primary schools (75% of all primary schools) implemented the Taste Lessons programme in the period of January 2017–June 2020 [32].
